# Biomechanical outcomes of superior capsular reconstruction for irreparable rotator cuff tears by different graft materials-a systematic review and meta-analysis

**DOI:** 10.3389/fsurg.2022.939096

**Published:** 2023-01-09

**Authors:** Xiaoxiong Zhao, Liang Wen, Bo Zhang, Jialin Jia

**Affiliations:** The Department of Orthopedics, Beijing Chaoyang Hospital, Capital Medical University, Beijing, China

**Keywords:** rotator cuff, superior capsular reconstruction, graft, material, biomechanics

## Abstract

**Background:**

Irreparable rotator cuff tears (IRCT) are defined as defects that cannot be repaired due to tendon retraction, fat infiltration, or muscle atrophy. One surgical remedy for IRCT is superior capsular reconstruction (SCR), which fixes graft materials between the larger tuberosity and the superior glenoid.

**Patients and methods:**

The Preferred Reporting Items for Systematic Reviews and Meta-Analysis (PRISMA) criteria were followed for conducting the systematic review and meta-analysis. From their inception until February 25, 2022, Pubmed, Embase, and Cochrane Library's electronic databases were searched. Studies using cadavers on SCR for IRCT were also included. The humeral head's superior translation and subacromial peak contact pressure were the primary outcomes. The humeral head's anteroposterior translation, the kind of graft material used, its size, and the deltoid load were the secondary outcomes.

**Results:**

After eliminating duplicates from the search results, 1,443 unique articles remained, and 20 papers were finally included in the quantitative research. In 14 investigations, the enhanced superior translation of the humeral head was documented in IRCTs. In 13 studies, a considerable improvement following SCR was found, especially when using fascia lata (FL), which could achieve more translation restraints than human dermal allograft (HDA) and long head of bicep tendon (LHBT). Six investigations reported a subacromial peak contact pressure increase in IRCTs, which could be rectified by SCR, and these studies found a substantial increase in this pressure. The results of the reduction in subacromial peak contact pressure remained consistent regardless of the graft material utilized for SCR. While there was a statistically significant difference in the change of graft material length between FL and HDA, the change in graft material thickness between FL and HDA was not significant. The humeral head's anterior-posterior translation was rising in IRCTs and could be returned to its original state with SCR. In five investigations, IRCTs caused a significant increase in deltoid force. Furthermore, only one study showed that SCR significantly decreased deltoid force.

**Conclusion:**

With IRCT, SCR might significantly decrease the glenohumeral joint's superior and anterior-posterior stability. Despite the risks for donor-site morbidity and the longer recovery time, FL is still the best current option for SCR.

## Introduction

Irreparable rotator cuff tears (IRCT) are defined as defects that cannot be completely repaired due to tendon retraction, fat infiltration, or muscle atrophy (1–4). These defects present a significant challenge to shoulder surgeons who attempt to fully repair the tearing tendons (5–7). Due to abnormal superior humeral head translation and narrowing of the subacromial space, IRCT may result in functional deficits and/or pain in patients (8–10), which can cause severe pain, loss of function, reduced range of motion, and affect patients' quality of life (11–14).

Recently, superior capsular reconstruction (SCR), which was first reported as a surgical alternative to IRCT by Mihata in 2013, has been used (15). The graft was utilized in this surgical surgery to adhere laterally to the larger tuberosity and medially to the superior glenoid. The graft's biomechanical function was as a static stabilizer that diminishes the pressure at the subacromial contact pressure and prevents superior translation of the humeral head (16, 17). As a new arthroscopic technique, the selection of graft materials was disputed (18–20). SCR with fascia lata(FL) showed a significant reduction in pain and functional improvements, such as range of motion, and rates of return to work and sports (21–23). Despite these good results, donor-site morbidity and prolonged surgical time concern many surgeons (24–26). Therefore, several alternative grafts have been applied, such as the long head of the bicep tendon(LHBT), human dermal allograft(HDA), xenograft, and synthetic graft materials (27–34). However, which graft material is optimal for SCR is still subject to debate.

Therefore, we conducted a systematic review and meta-analysis to:

(1) review the literature regarding biomechanical outcomes of SCR for IRCT; (2) compare the biomechanical outcomes of various grafts used in SCR.

## Materials and methods

The Preferred Reporting Items for Systematic Reviews and Meta-Analysis (PRISMA) standards were followed in conducting the systematic review and meta-analysis (35). The review followed the methods recommended by the Cochrane Handbook for Systematic Reviews of Interventions (36). There has never been a protocol for systematic reviews or meta-analyses, as far as we are aware.

### Eligibility criteria

Research was taken into consideration for review if it met the following inclusion criteria:

• Cadaveric study
• Undergoing SCR for IRCT
• Reporting biomechanical outcomes: The primary outcomes were superior translation of humeral head and subacromial peak contact pressure. Secondary outcomes included anteroposterior translation of humeral head, the type of grafts, the side of grafts and deltoid load.

Exclusion criteria:

• Studies involving animals or operative techniques
• Duplicates and relevant research
• Case reports
• Full text not available
• Non-English articles

### Search strategy

From their inception until February 25, 2022, the electronic databases of Pubmed, Embase, and Cochrane Library were searched. The terms “"Superior capsular reconstruction” OR “superior capsule reconstruction” OR “irreparable rotator cuff"” were utilized as the search strategy and appeared in the title, abstract, or keywords fields. In case any were overlooked by the initial search, all references in the included studies were cross-referenced for inclusion by two authors (ZX and JJ).

### Trial selection

After duplicates were eliminated, all records' titles and abstracts were initially reviewed by ZX and JJ. Each potentially suitable article's whole text was examined, and disagreements were settled by a third independent reviewer (ZB).

### Data extraction

Two reviewers (ZX and JJ) separately compiled trial information (year of publication, nation of origin, and number of samples), intervention and control features, as well as primary and secondary outcome data, by using a standardized data extraction form. When more information was necessary, trial authors were contacted.

### Risk of bias assessment

Potential publication bias was examined using contour enhanced funnel plots and Egger's regression test using RStudio software version 2021.09.2(R Foundation for Statistical Computing, Vienna, Austria).

Methodological risk of bias of studies was performed through a checklist proposed by Towns and Black (37). The recommended scoring criteria was maintained, resulting in a total of 26 items with a possible maximum score range of 0 to 26, with higher scores indicating a reduced risk of bias.

### Statistical analysis

All subjects' and outcome parameters' weighted means and standard deviations were computed for continuous data. By comparing to the IRCT as a shared control, standard mean differences between the SCR groups and the intact rotator cuff groups were calculated.

All statistical analyses were conducted by the Meta package as part of RStudio software version 2021.09.2(R Foundation for Statistical Computing, Vienna, Austria). A random effects model was used in a meta-analysis to aggregate outcome measures from various studies. As summary statistics, relative risks (RR) with matching 95% confidence intervals were applied. For superior translation of the humeral head, subacromial peak contact pressure, graft material size, and deltoid load, forest plots were made. The various grafts were to be the subject of a subgroup analysis. If the *I*^2^ statistic was higher than 50% or the *p*-value for the Chi2 statistic was less than 0.05, statistical heterogeneity was deemed to be significant. Results of individual trials were presented in cases where meta-analysis was not feasible.

## Results

**Figure 1:**
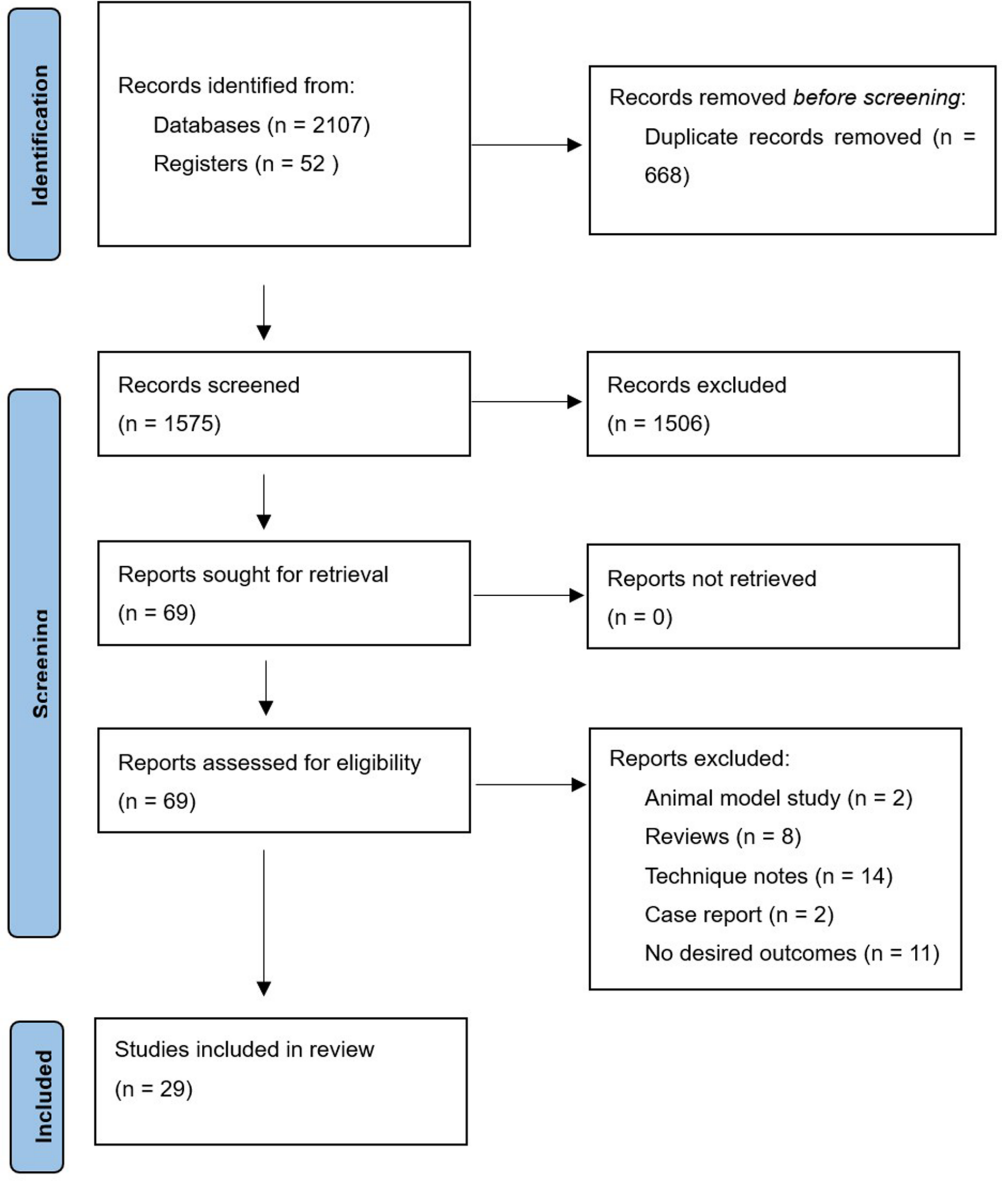
Flow diagram of literature processing.

After duplicates were removed, the initial search produced 1,575 original items, of which 69 were thought to be potentially eligible. In the final analysis, 29 cadaveric investigations were considered (8, 15, 17, 21, 27, 38–61). Figure 1 illustrates the flow diagram.

Characteristics of included trials are summarized in Table 1. The 29 trials that were featured were published between 2012 and 2022. The listed studies' sample sizes range from 5 to 15 people. Every study made use of dissected, soft tissue-free cadaveric shoulders for the shoulders. The capsule, coracoacromial ligament, four rotator cuff tendons, pectoralis major tendinous insertion, latissimus dorsi, and three deltoid heads were all still there. However, the loading conditions used in these investigations varied significantly. Of these, 20 studies achieved the balanced system: deltoid, 40N; pectoralis major, 20N; latissimus dorsi, 20N; supraspinatus, 10N; subscapularis, 10N; infraspinatus, 5N; teres minor, 5N. The unbalanced loading condition was achieved by removing the loads from latissimus dorsi and pectoralis major, and added an additional 40N to the deltoid. Two studies reduced deltoid load by half (balanced system: 20N; unbalanced system: 40N) (49, 52). The loading conditions were not properly displayed in the other six experiments (46–48, 51, 53, 56). Therefore, only 20 studies were involved in the quantitative research (8, 15, 17, 21, 27, 38–45, 50, 54, 55, 57–60).

**Table 1:** Study characteristics of studies included in the review.

Year	Lead Author	Shoulders	Graft Materials	Loading Conditions
2021	Tibone	6	HDA	Condition 1
2021	Shah	8	FL	Condition 1
2021	Lacheta	12	HDA	Condition 1
2021	Lobao	8	HDA	Condition 1
2021	Denard	8	LHBT	Condition 1
2021	Denard	8	Semitend	Condition 1
2021	Berthold	10	Semitend	Unknown
2021	Berthold	8	LHBT	Unknown
2020	Vredenburgh	8	FL	Condition 1
2020	Smith	5	HDA	Condition 1
2020	Rybalko	6	HDA	Condition 2
2020	Reeves	8	Balloon	Condition 1
2020	Han	8	LHBT	Condition 1
2020	Dyrna	12	HDA	Unknown
2020	Curtis	8	HDA	Condition 2
2020	Cline	8	FL	Condition 1
2020	Adams	15	HDA	Unknown
2019	Singh	8	Balloon	Condition 1
2019	Scheiderer	8	HDA	Unknown
2019	Omid	8	HDA	Condition 1
2019	Lobao	14	Balloon	Unknown
2019	Leschinger	6	FL	Condition 1
2019	Han	7	LHBT	Condition 1
2019	Croom	8	PT	Condition 1
2018	Park	9	LHBT	Condition 1
2017	Mihata	8	FL	Condition 1
2016	Mihata	8	FL	Condition 1
2012	Mihata	8	FL	Condition 1

FL: fascia lata; HDA: human dermal allograft; LHBT: long head of bicep tendon; PT: patellar tendon; Semitend: semitendinosus.

Condition 1: balanced system: deltoid, 40N; pectoralis major, 20N; latissimus dorsi, 20N; supraspinatus, 10N; subscapularis, 10N; infraspinatus, 5N; teres minor, 5N. Unbalanced loading condition was achieved by removing the loads from latissimus dorsi and pectoralis major and added an additional 40N to the deltoid.

Condition 2: balanced system: deltoid, 20N; pectoralis major, 10N; latissimus dorsi, 10N; supraspinatus, 5N; subscapularis, 5N; infraspinatus, 2.5N; teres minor, 2.5N. Unbalanced loading condition was achieved by removing the loads from latissimus dorsi and pectoralis major and added an additional 20N to the deltoid.

### Superior translation of humeral head

**Figure 2:**
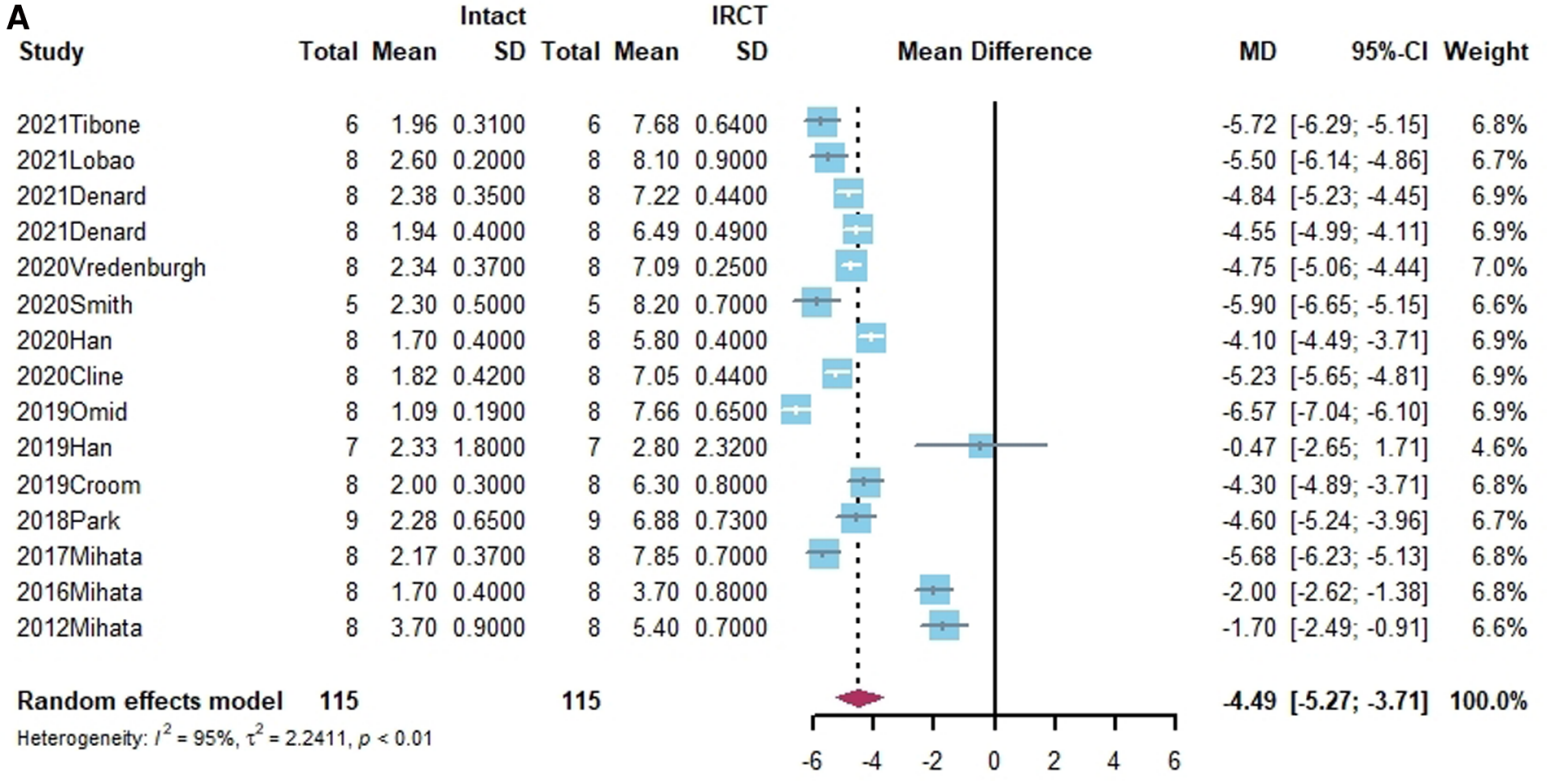
(**A**) forest plot comparing the mean difference (MD) of superior translation of humeral head between intact cuff and IRCT. (**B**) Forest plot comparing the mean difference (MD) of superior translation of humeral head between IRCT and SCR. (**C**) Forest plot comparing the mean difference (MD) of superior translation of humeral head between Intact cuff and SCR.

Superior translation of humeral head was reported in 15 studies (15, 17, 21, 27, 38, 40, 41, 43–45, 50, 55, 58–60). In 14 studies, a significant increase was found in superior translation of the humeral head after establishing an irreparable rotator cuff tear, except in the Han study (27). In addition, 13 studies—all except the Denard study (45)— found a significant improvement in the superior translation of the humeral head following SCR. Of these, 10 studies reported that superior translation could be restored to intact rotator cuff by SCR (15, 17, 21, 27, 40, 41, 44, 55, 58, 60). One study reported lower superior translation after SCR, compared to intact rotator cuff (59). The standard mean difference between intact rotator cuff and IRCT was −4.49 (95% CI [−5.27;-3.71], *I*^2 ^= 95%, *p* < 0.01). For SCR vs. intact and IRCT vs. SCR, the standard mean difference was −1.14 (SCR vs. intact, 95% CI [−2.07;−0.22], *I*^2 ^= 99%, *p* < 0.01) and 3.33 (IRCT vs. SCR, 95% CI [2.434;4.33], *I*^2 ^= 98%, *p* < 0.01), respectively (Figures 2A–C).

**Figure:**
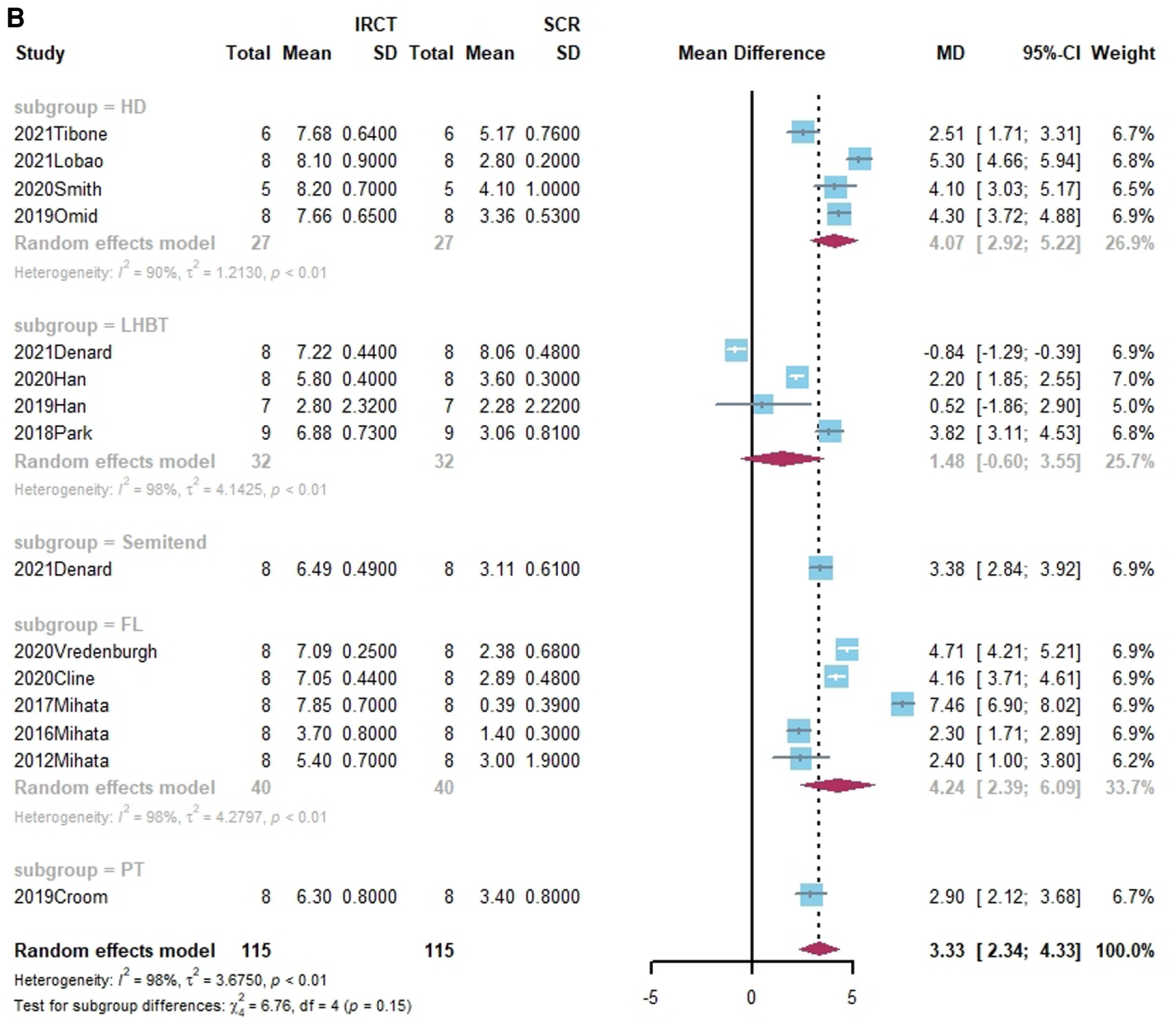


**Figure:**
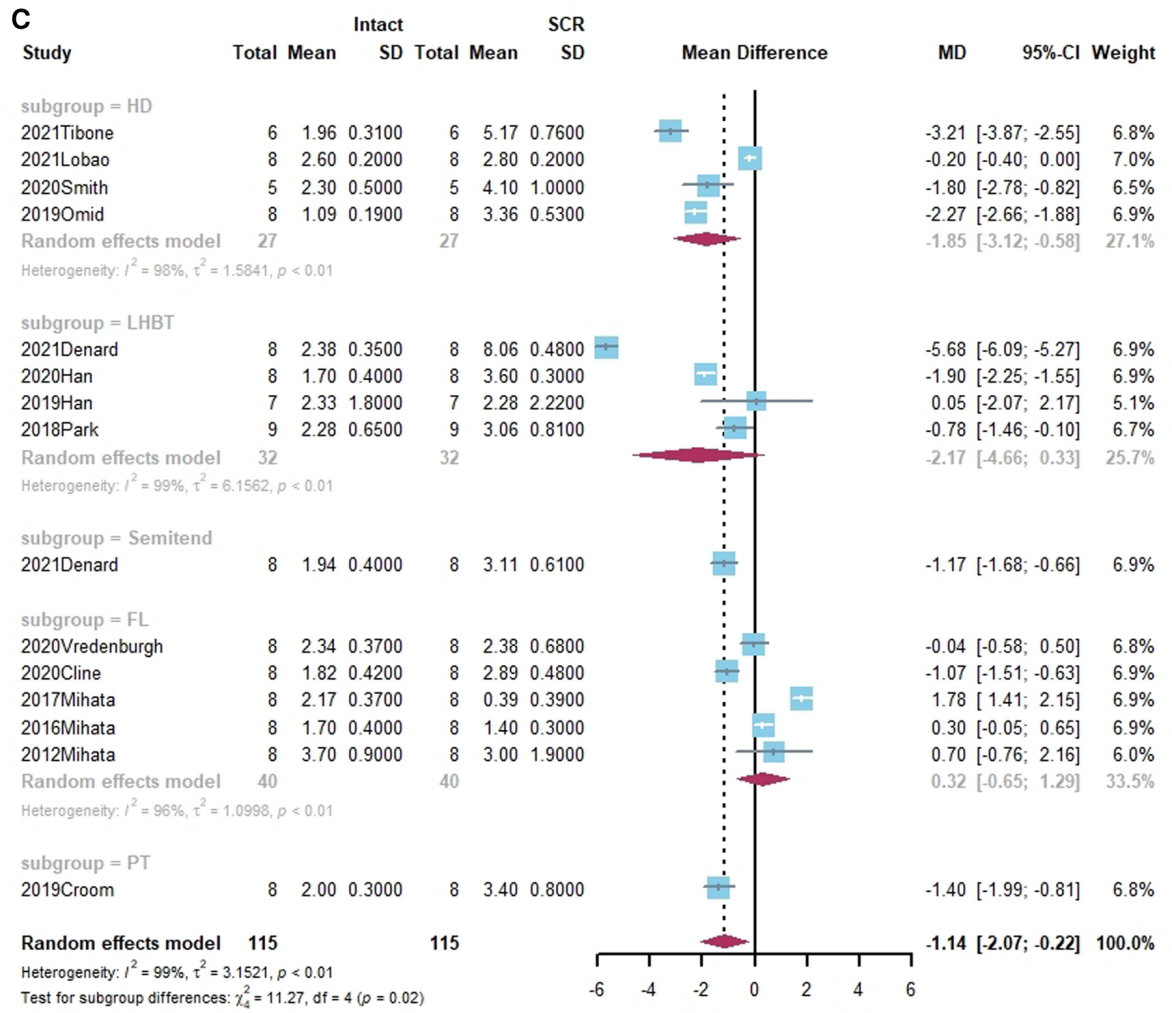


In a subgroup analysis, SCR with FL achieved superior translation more closed to Intact cuff (MD = 0.32, 95% CI [-0.65;1.29]). The superior translation after SCR with HDA or LHBT was slightly greater than Intact cuff (HD: MD = −1.85, 95% CI [−3.12;−0.58]; LHBT: MD = −2.17, 95% CI [−4.66;0.33]). Meanwhile, the difference between FL and other grafts was statistically significant (X^2 ^= 11.27, *p* = 0.02).

### Subacromial peak contact pressure

**Figure 3:**
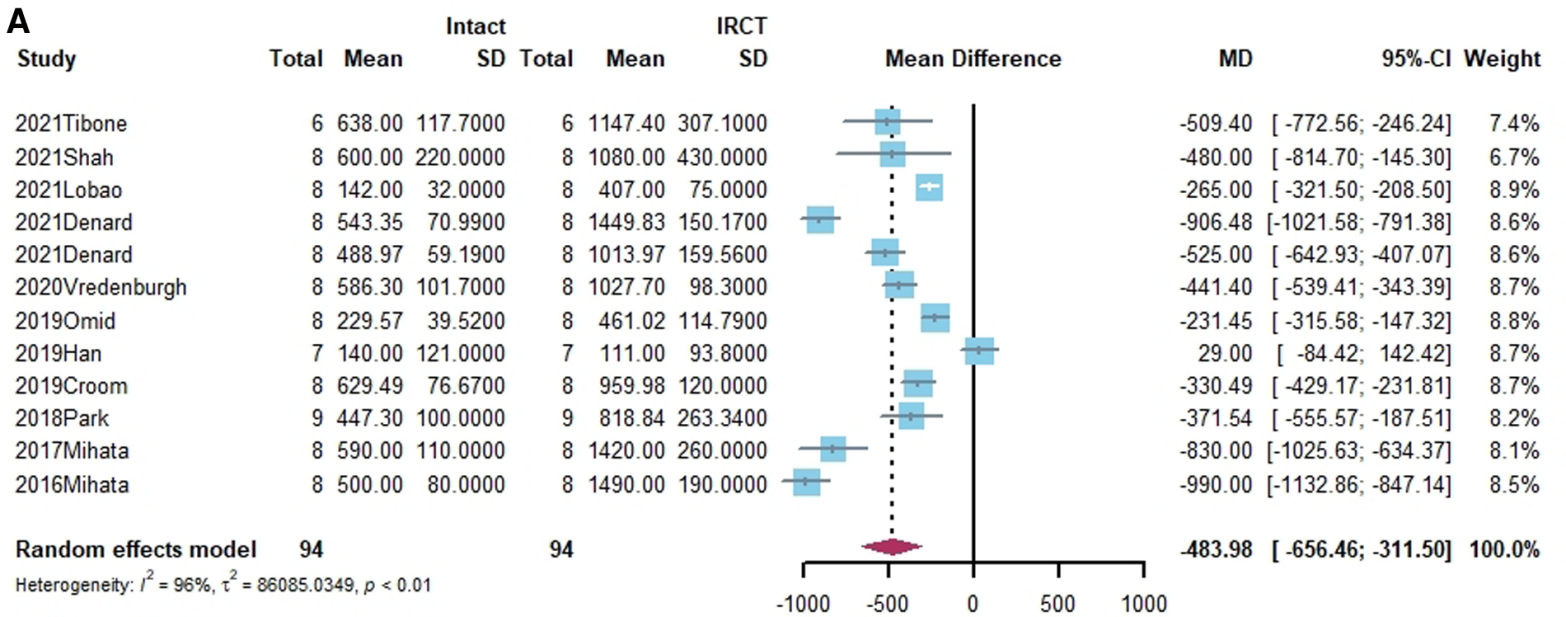
(**A**) Forest plot comparing the mean difference (MD) of subacromial peak contact pressure between Intact cuff and IRCT. (**B**) Forest plot comparing the mean difference (MD) of subacromial peak contact pressure between IRCT and SCR. (**C**) Forest plot comparing the mean difference (MD) of subacromial peak contact pressure between Intact cuff and SCR.

Subacromial peak contact pressure was reported in 12 studies (21, 27, 38–41, 44, 45, 55, 58–60). Of these, six studies noted a marked increase in subacromial peak contact pressure following the appearance of the irreparable rotator cuff tear (21, 39–41, 59, 60). All studies restored subacromial peak contact after SCR. The standard mean difference among intact rotator cuff, IRCT, and SCR was −483.98 (Intact vs. IRCT, 95% CI [−656.46;−311.5], *I*^2 ^= 96%, *p* < 0.01), 37.15 (Intact vs. SCR, 95% CI [-145.56; 219.85], *I*^2 ^= 98%, *p* < 0.01) and 519.28 (IRCRT vs. SCR, 95% CI [298.83;739.73], *I*^2 ^= 98%, *p* < 0.01) respectively (Figures 3A–C).

**Figure:**
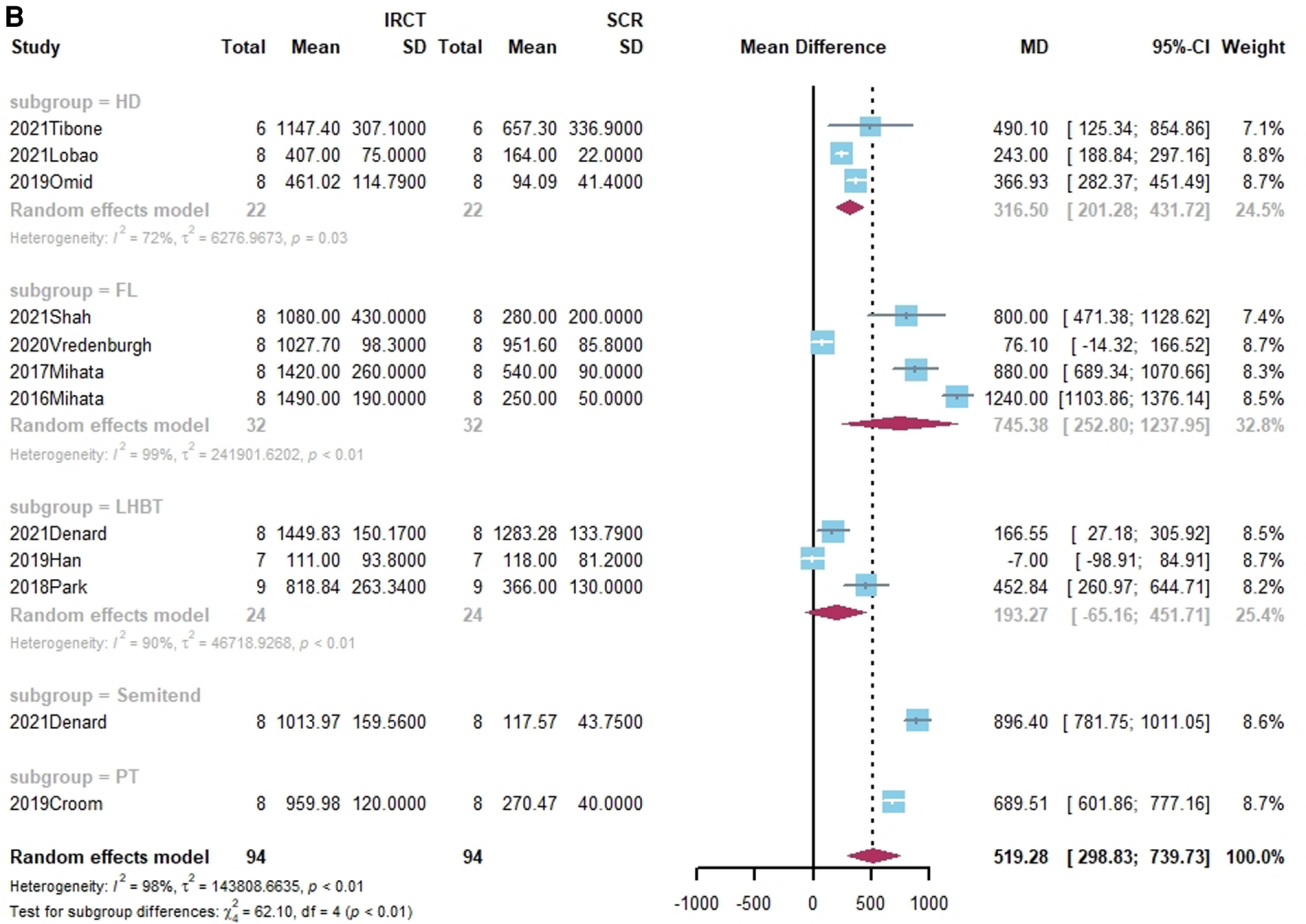


**Figure:**
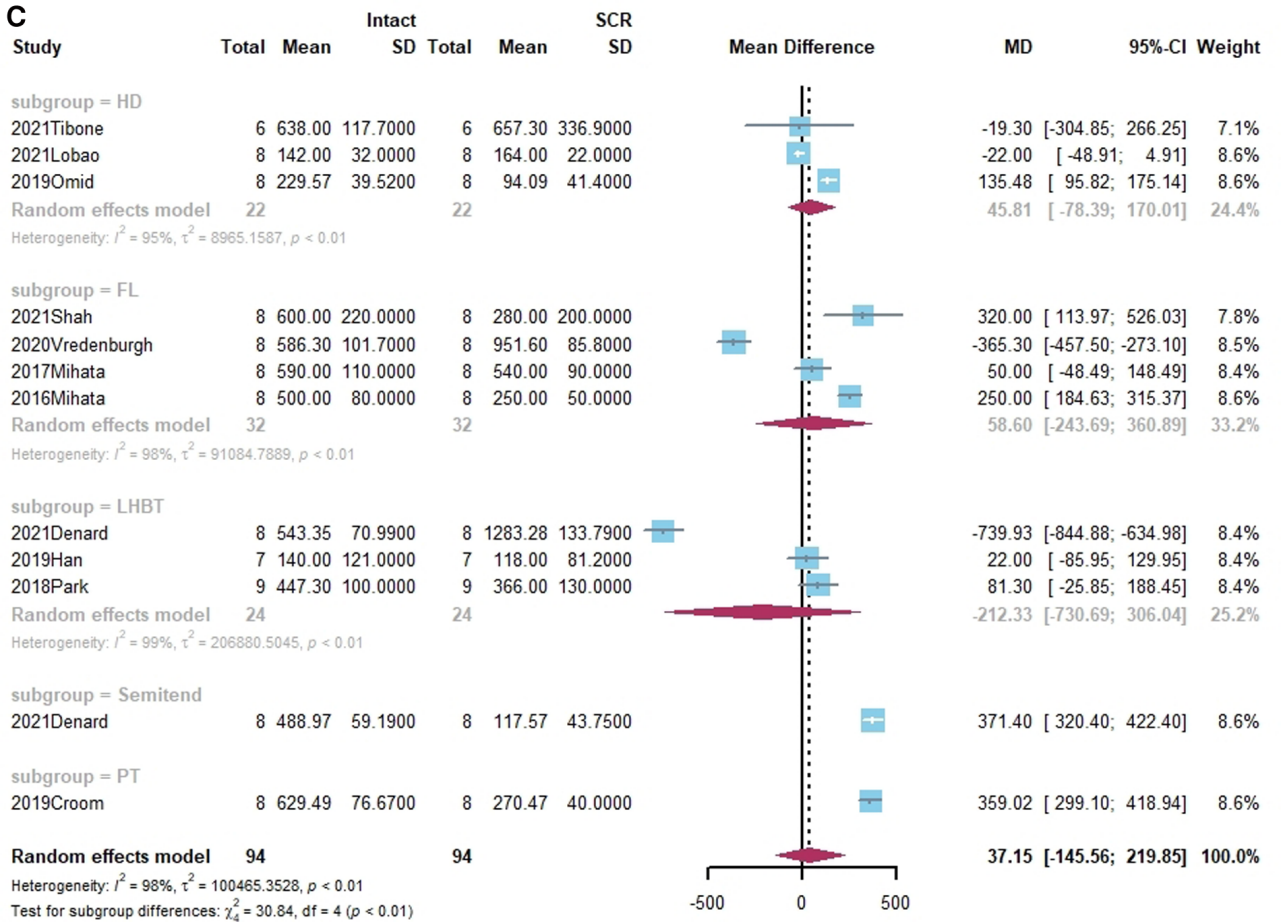


According to a subgroup analysis, the subacromial peak contact pressure may be brought back to the same level as the intact cuff regardless of the type of grafts utilized for SCR.

### The size of grafts

**Figure 4:**
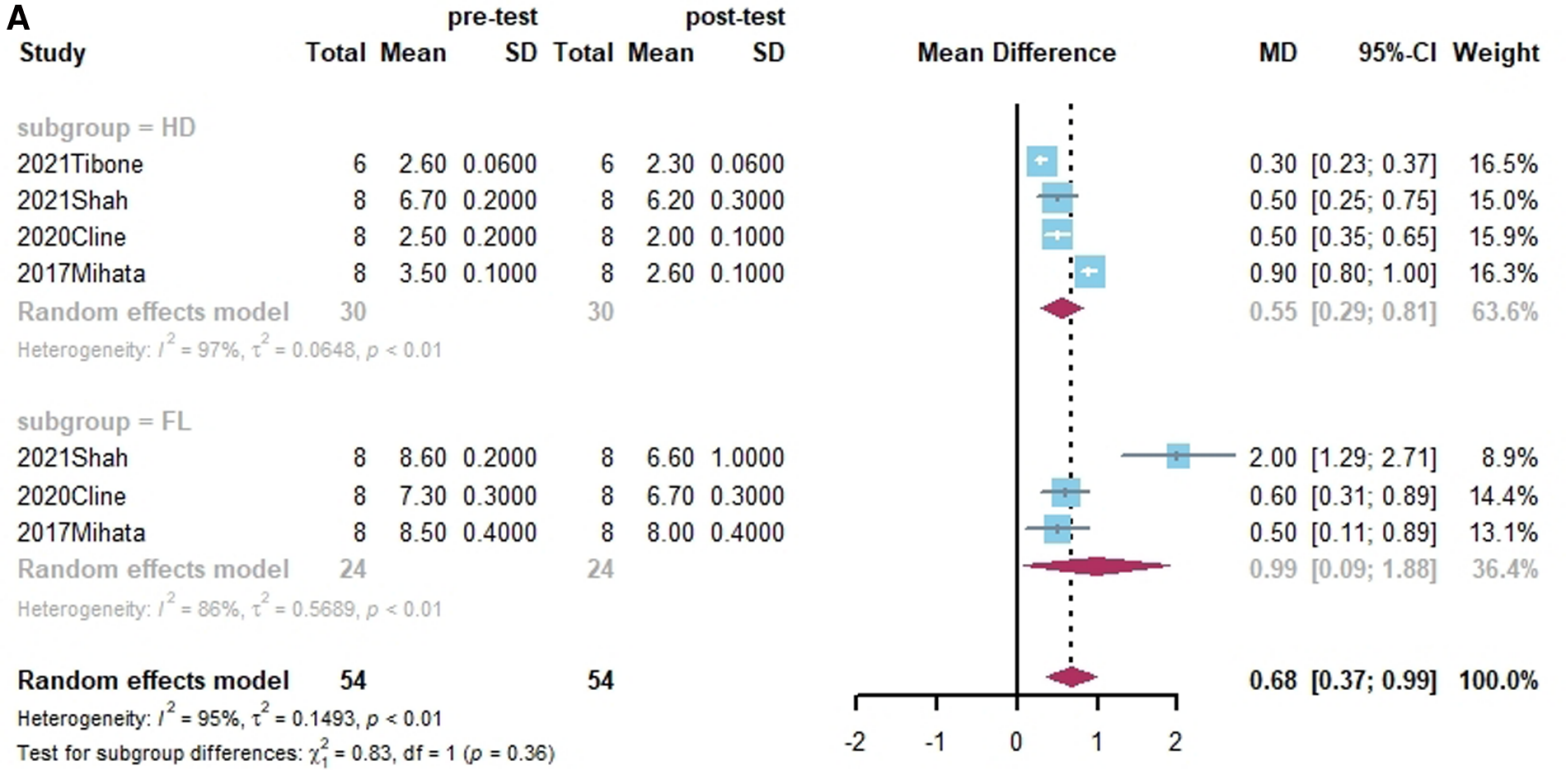
(**A**) Forest plot comparing the mean difference (MD) of thickness of graft materials in pre- and post-test. (**B**) Forest plot comparing the mean difference (MD) of length of graft materials in pre- and post-test.

Four studies reported on the size of grafts (38, 39, 43, 59). Of these, three studies presented both grafts thickness and lengths (39, 43, 59). In a subgroup analysis, there was no statistically significant difference between HDA and FL in terms of the change in graft thickness. (x^2 ^= 0.83, *p* = 0.36). While the change in length of FL was substantially smaller than that of HD in terms of grafts (x^2 ^= 17.87, *p* < 0.01). Shown in Figures 4A,B.

**Figure:**
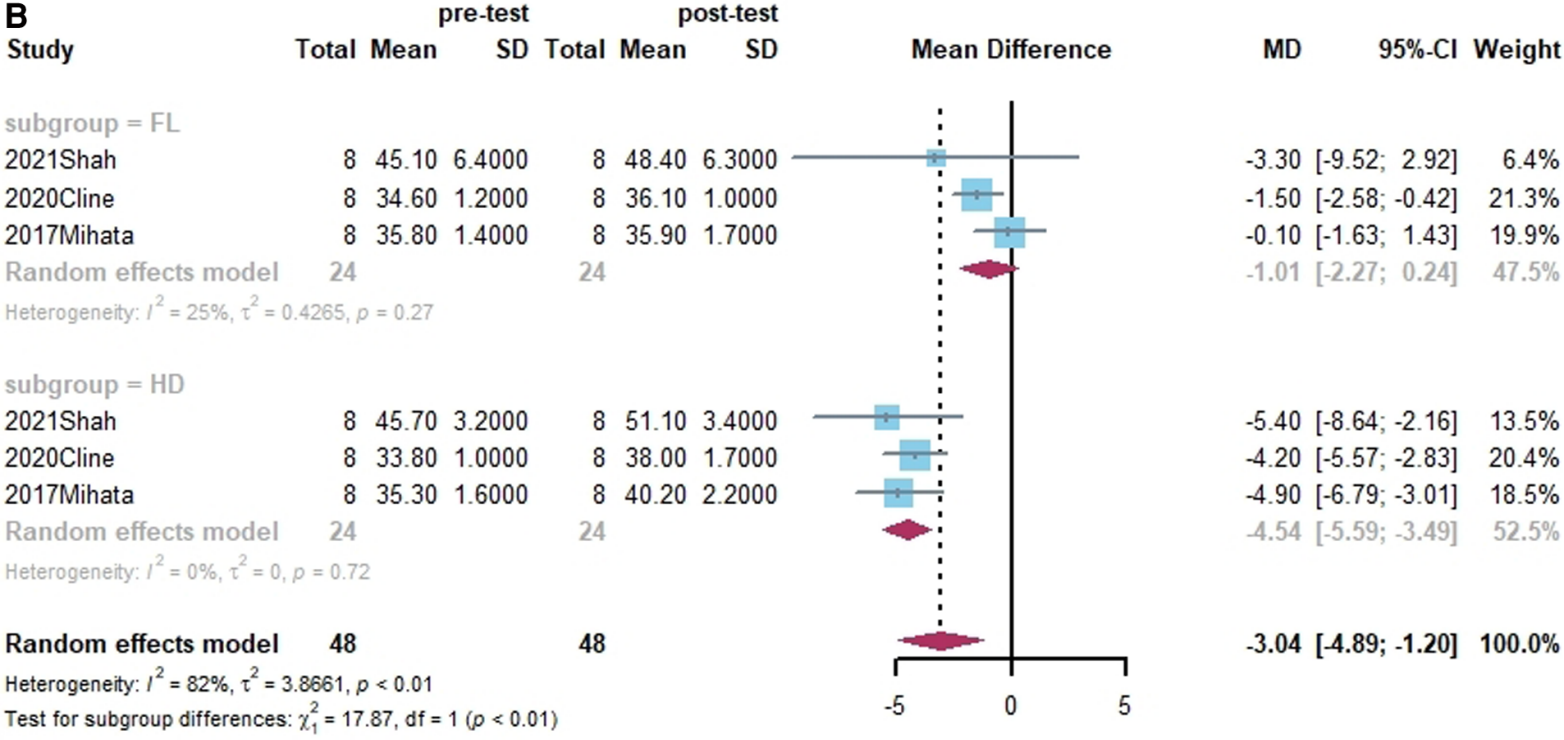


### Anteroposterior translation of humeral head

**Figure 5:**
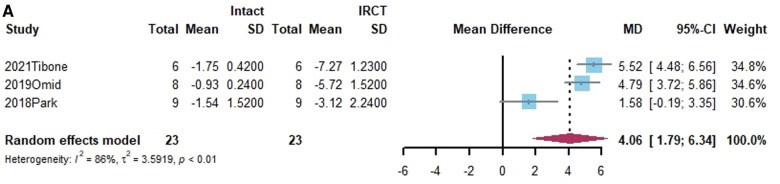
(**A**) Forest plot comparing the mean difference (MD) of antero-posterior translation of humeral head between Intact cuff and IRCT. (**B**) Forest plot comparing the mean difference (MD) of antero-posterior translation of humeral head between IRCT and SCR. (**C**) Forest plot comparing the mean difference (MD) of antero-posterior translation of humeral head between Intact cuff and SCR.

Anteroposterior translation of the humeral head was described in four studies overall (38, 40, 41, 55). Of these, three studies found that, following the occurrence of the irreparable rotator cuff tear, the posterior translation of humeral head was significantly increased, and could be returned to the level of Intact cuff after SCR (38, 41, 55). Shown in Figures 5A–C.

**Figure:**
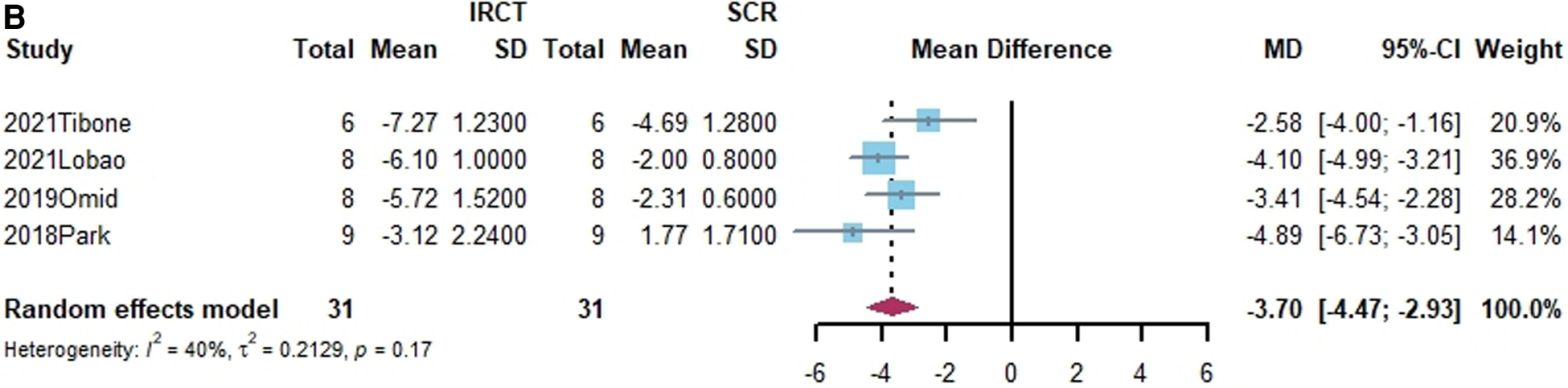


**Figure:**
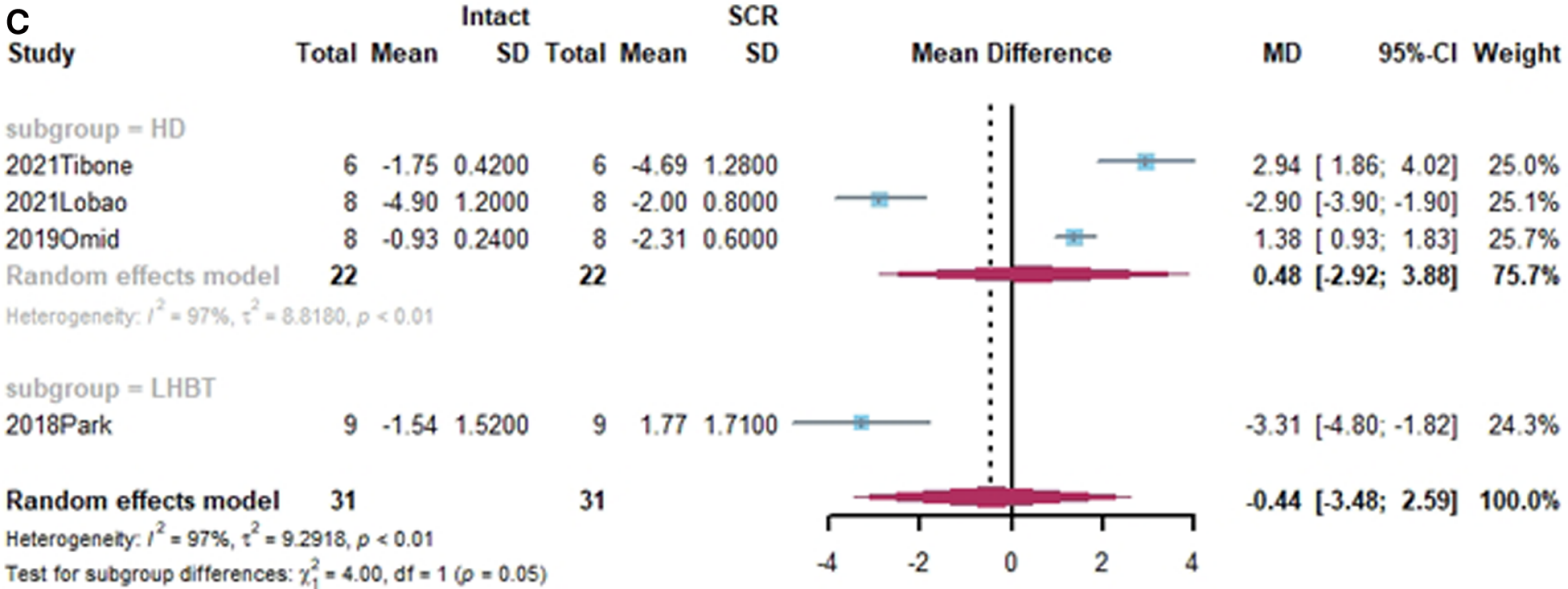


### Deltoid force

**Figure 6:**
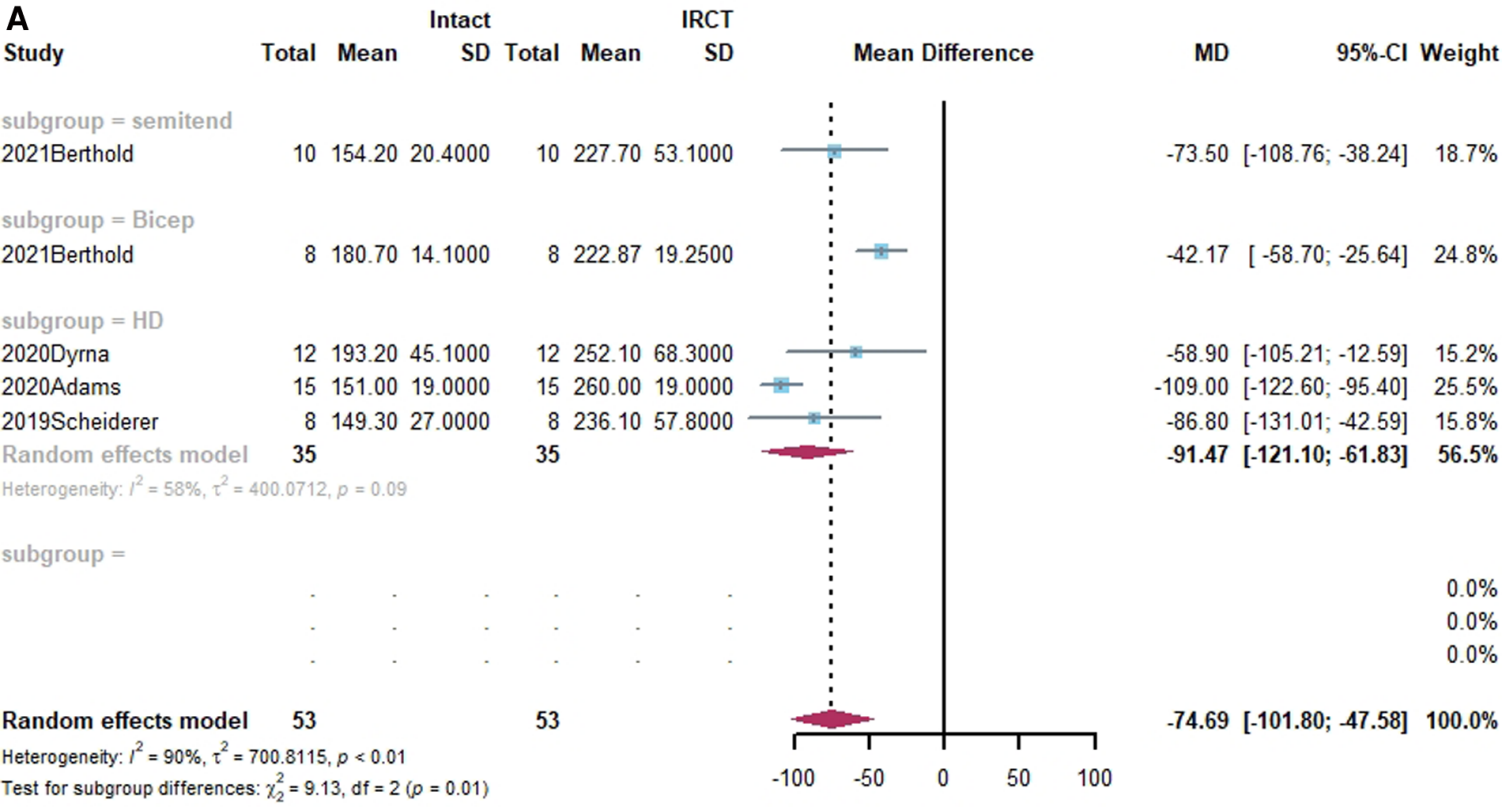
(**A**) Forest plot comparing the mean difference (MD) of deltoid force between Intact cuff and IRCT. (**B**) Forest plot comparing the mean difference (MD) of deltoid force between IRCT and SCR. (**C**) Forest plot comparing the mean difference (MD) of deltoid force between Intact cuff and SCR.

Five studies reported on deltoid force (46–48, 51, 53). Because the loading circumstances were not disclosed in any of the five studies, a qualitative analysis was done. In all five studies, the establishment of the IRCT resulted in a considerable increase in deltoid force relative to the intact cuff. Only one study found that deltoid force had considerably improved following SCR (46). Meanwhile, all studies reported that deltoid force after SCR was not as low as that of intact cuff (Figures 6A–C).

**Figure:**
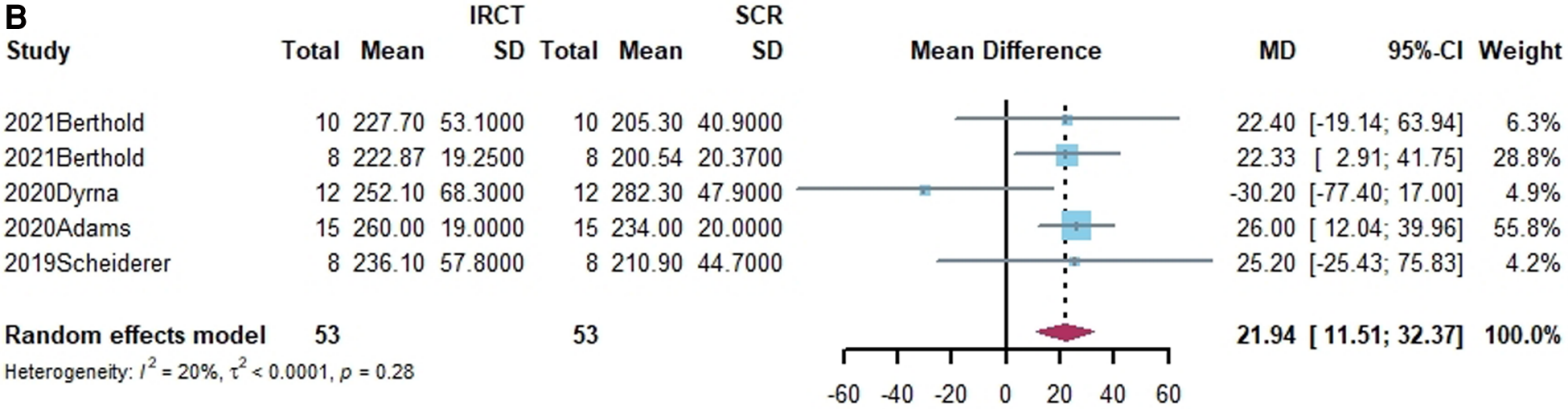


**Figure:**
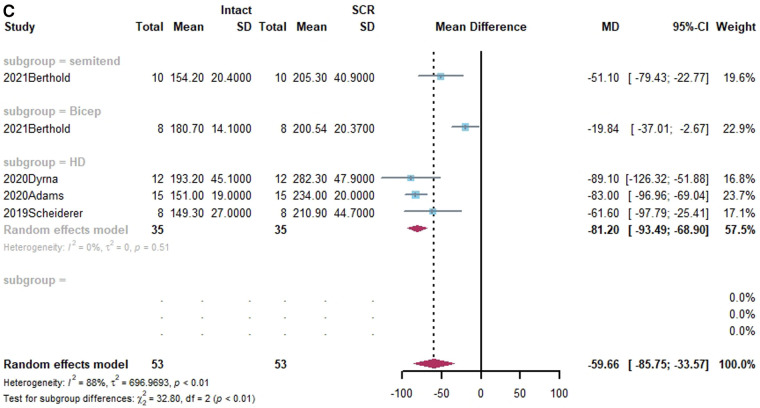


### Risk of publication bias

**Figure 7:**
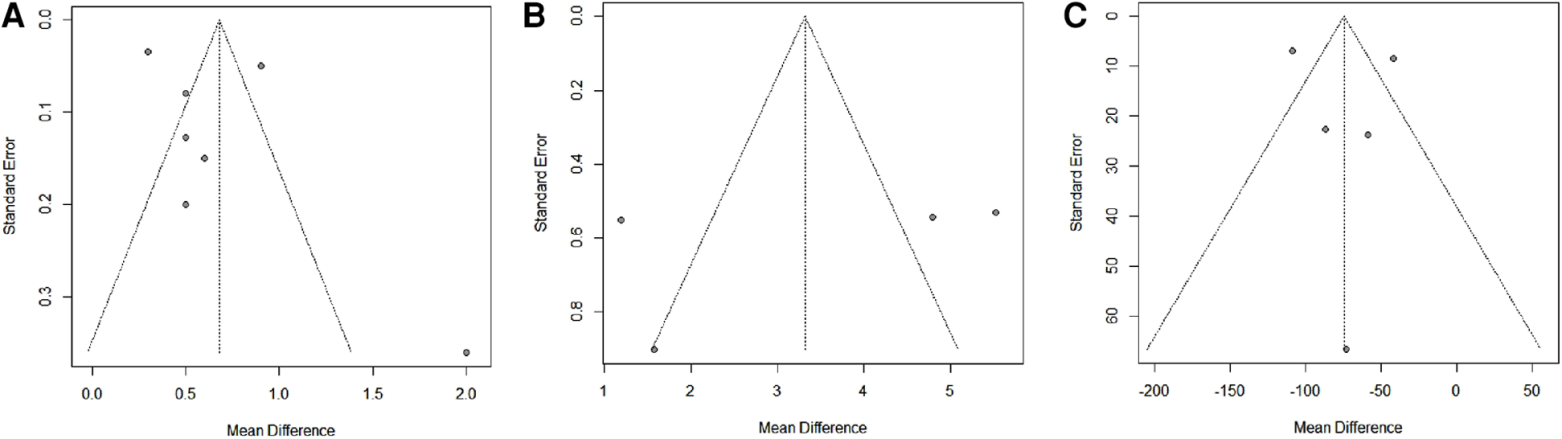
The funnel plots for the size of grafts (**A**), anteroposterior translation of humeral head (**B**), and deltoid force (**C**).

Egger's regression test demonstrated no evidence of publication bias for superior translation of humeral head (*t* = 1.01, *p* = 0.329), and subacromial peak contact pressure (*t* = −1.53, *p* = 0.157). The funnel plots demonstrated no evidence of publication bias for the size of grafts, anteroposterior translation of humeral head, and deltoid force. The funnel plots are shown in Figure 7.

### Risk of methodological bias

Out of a possible maximum of 26 points, the mean risk of bias score across studies was 12.03, SD = 1.68 (range = 9 to 14). Lack of blind procedure was common in these studies.

## Discussion

In this systematic review and meta-analysis, we discovered that SCR with FL, in comparison to HDA and LHBT, achieved better results in terms of restricting superior translation of humeral head and graft deformation. However, all of the studies included in this meta-analysis used cadavers, which is the IV-level of evidence-based medicine.

The large majority of studies showed that IRCT increased the superior translation of the humeral head, which suggests that the superior capsule, along with dynamic strengthening of the rotator cuff, is an essential static stabilizer that prevents superior translation of the humeral head (10, 62). SCR with FL, however, was able to achieve a level of humeral head translation that was closer to that of an intact cuff when compared to SCR with HDA and LHBT, and this may be due to the different graft thickness (43, 48, 60, 63). It is crucial to remember that the improved translation was not restored to its original state regardless of the graft materials employed for SCR.

In line with our findings, IRCT may also cause subacromial impingement by raising the subacromial contact pressure. Subacromial contact pressure was significantly reduced after SCR and nearly returned to its original level. With these two enhancements, SCR for IRCT could improve the functional outcomes and restore the biomechanical outcomes, as seen in the clinical studies (39, 45, 58, 59).

The stability of the glenohumeral joint, which is crucial for shoulder function and elevation, is indicated by the humeral head's anteroposterior location within the glenoid (64, 65). In our study, the humeral head was seen to move posteriorly following IRCT. SCR was able to center the humeral head in the glenoid and successfully restore the integrity of the capsule. Therefore, by recreating concavity compression, the humeral head might be fixed to the glenoid.

One of the most crucial elements affecting SCR is graft. Since SCR was used, numerous grafts have been developed for greater biomechanical properties and lower prices. The most frequent are LHBT, HDA, and FL grafts. According to our research, HDA is less effective than FL at limiting the superior translation of the humeral head at the same graft fixation angle. Additionally, SCR with LHBT had less effect on limiting humeral head superior translation as compared to FL and HDA, which may be related to the LHBT's insufficient thickness and flatness, which, in turn, may be a factor in the “spacer” effects. Additionally, during the same mechanical test cycles, HDA deformed more than FL, which explains why SCR will eventually fail.

Makovicka et al. (20) conducted a systematic review of biomechanical outcomes by graft type. However, this study only included eight studies, and five of these were conducted by the same author. Besides, only comparison between fascia lata allograft and dermal allograft was analyzed. Our study is the first systematic evaluation and meta-analysis of several grafts for SCR that we are aware of. Biomechanical studies illustrated that FL has the highest biomechanical efficiency when compared to HDA and LHBT, which serves as a reminder that new graft materials need to be investigated since they need to be less expensive, more robust, and more biocompatible. Following the biomechanical outcomes, LHBT was shown not to be a suitable graft for SCR, although there are various surgical procedures that use LHBT. The limitations of this review relate to: (1) In our study, the effectiveness of each graft material was examined only from the perspective of biomechanics; clinical results were not taken into account. However, we thought that strong graft biomechanics was a prerequisite for good clinical outcomes; (2) The studies that were involved used various measuring techniques, which might have impacted the baseline of the measured data. However, the comparison of the data before and after the experiments was unaffected by this difference; (3) The surgical method is another element that affects SCR in addition to the graft. We did not group the various operations in our study according to the surgical methods. However, the surgical procedure had no impact on the graft's biomechanical effectiveness.

## Conclusion

In conclusion, SCR combined with IRCT could greatly increase the superior and anteroposterior stability of the glenohumeral joint. However, it should be highlighted that SCR, particularly SCR with LHBT or HDA, might not restore the glenohumeral joint's original biomechanical condition. Despite the potential for donor-site morbidity and the longer recovery time, FL is still the best current option for SCRe. The goal of future research should be to discover new grafts that are less expensive, more effective from a biomechanical standpoint, and have good biocompatibility for SCR.

## Data Availability

The original contributions presented in the study are included in the article/Supplementary Material, further inquiries can be directed to the corresponding author/s.
