# Corrigendum: Biomechanical outcomes of superior capsular reconstruction for irreparable rotator cuff tears by different graft materials-a systematic review and meta-analysis

**DOI:** 10.3389/fsurg.2023.1173516

**Published:** 2023-05-22

**Authors:** Xiaoxiong Zhao, Liang Wen, Bo Zhang, Jialin Jia

**Affiliations:** The Department of Orthopedics, Beijing Chaoyang Hospital, Capital Medical University, Beijing, China

**Keywords:** rotator cuff, superior capsular reconstruction, graft, material, biomechanics

A Corrigendum on Biomechanical outcomes of superior capsular reconstruction for irreparable rotator cuff tears by different graft materials-a systematic review and meta-analysis Zhao X, Jia J, Wen L and Zhang B. (2023). Front. Surg. 9:939096. doi: 10.3389/fsurg.2022.939096.

Author List

In the published article, there was an error in the author list, and author Bo Zhang, Liang Wen and Jialin Jia was erroneously sorted. The corrected author list appears below.

Xiaoxiong Zhao*, Liang Wen, Bo Zhang, Jialin Jia

Correspondence

The author Bo Zhang was erroneously included as the correspondence.

Xiaoxiong Zhao is the new correspondence author, and his email address is zhaoxiaoxiong@ccmu.edu.cn.

The authors apologize for this error and state that this does not change the scientific conclusions of the article in any way. The original article has been updated.

